# Development and validation of a nomogram for predicting unplanned PICC removal in preterm infants with gestational age <32 weeks

**DOI:** 10.3389/fped.2026.1725396

**Published:** 2026-02-12

**Authors:** Yuedi Hu, Yu Lang, Leilei Shen, Ling Yan

**Affiliations:** Department of Pediatrics, Third Military Medical University Southwest Hospital, Chongqing, China

**Keywords:** peripherally inserted central catheters, prediction nomogram, preterm infants, risk prediction, unplanned removal

## Abstract

**Objective:**

To develop and validate a risk prediction model for unplanned removal (UR) of peripherally inserted central catheters (PICC) in preterm infants with gestational age (GA) < 32 Weeks.

**Methods:**

This retrospective study analyzed preterm infants with PICC admitted to a neonatal intensive care unit (NICU) (January 2018 to December 2024). Clinical and catheter-related variables were assessed. Multivariable logistic regression identified predictors of PICC-UR, with model performance evaluated by C-index, calibration, and decision curve analysis (internal validation via 1000 bootstraps).

**Results:**

We identified five independent predictors for PICC-UR: insertion site (categorical), white blood cell count (WBC), platelet count (PLT), and fibrinogen (Fib) (all modeled as continuous linear terms), along with hypercholanemia (HCA). These predictors were integrated into a nomogram designed to estimate the individual risk of PICC-UR in preterm infants. The predictive model demonstrated a high accuracy with a C-index of 0.827 [95% confidence interval (CI): 0.740–0.915]. Internal validation confirmed excellent calibration and significant clinical utility based on decision curve analysis.

**Conclusions:**

This validated nomogram, incorporating insertion site, WBC, PLT, Fib and HCA, aids early identification of high-risk infants. It offers actionable insights for optimizing PICC fixation and biochemical monitoring, potentially reducing PICC-UR in NICU.

## 1 Background

The care of very (28 to <32 weeks) and extremely (<28 weeks) preterm infants remains a major neonatal challenge due to profound organ immaturity and physiological vulnerability (1). These infants often require prolonged secure intravenous access for parenteral nutrition, critical medications, and blood sampling, given their underdeveloped gastrointestinal/renal systems, high metabolic demands, and comorbidities (2–4). Conventional peripheral access is often inadequate due to fragile vasculature, skin compromise, and repeated puncture risks (5). Thus, peripherally inserted central catheters (PICC) have become indispensable, as they enable the delivery of life-sustaining therapies, thereby significantly contributing to care quality.

PICC, inserted via peripheral veins with tips terminating in central veins, enable safe infusion of hyperosmolar solutions (e.g., 20% lipid emulsions) and minimize trauma from repeated venipuncture. They are now the primary long-term vascular access for extremely preterm infants, used in >60% of cases with an average indwelling time of 19–57 days (6, 7). However, their widespread adoption has introduced unplanned removal (UR) as a critical complication, leading to repeated punctures, catheter-related bloodstream infections, increased costs, and potential treatment disruptions or exacerbation of clinical conditions. UR necessitates repeated vascular access procedures, causing patient discomfort, increasing the risk of infection, delaying treatment, and raising healthcare costs.

Despite progress in neonatal vascular access, risk factors for PICC-UR in preterm infants remain poorly characterized. We analyzed 257 preterm infants with PICC to identify clinical and laboratory predictors of PICC-UR, with the goal of developing a predictive nomogram for early risk stratification and targeted interventions to improve PICC retention and outcomes in this vulnerable population.

## 2 Materials and methods

### 2.1 Study subjects

We conducted a retrospective analysis of clinical data from preterm infants with gestational age (GA) < 32 weeks who underwent PICC placement in the neonatal intensive care unit (NICU) between January 2018 and December 2024. To avoid clustering effects, our analysis focused on the first PICC insertion per infant for those who underwent multiple PICC placements. In accordance with our unit's standard protocol for preterm infants of this GA, a single type of catheter (1.9 Fr single-lumen polyurethane catheter) was used for all insertions in this cohort. Inclusion criteria were: (1) GA <32 weeks; (2) first PICC placement during NICU stay. Only PICCs with confirmed optimal tip location (distal superior vena cava or inferior vena cava) verified by post-insertion x-ray were included in the study. Based on the occurrence of unplanned removal (UR) during the first PICC insertion, 55 preterm infants were classified into the UR group, while the remaining 202 comprised the non-UR group. Exclusion criteria included: (1) death prior to the planned removal of the PICC; (2) withdrawal of life-sustaining treatment or discharge against medical advice; (3) incomplete clinical data. Infants who died prior to planned PICC removal were excluded to avoid confounding, as the physiological state preceding death could profoundly influence both the studied laboratory parameters and the catheter's status, and the removal event in these circumstances does not represent the preventable outcome this study aims to predict.

Criteria for PICC-UR, based on previous studies, were as follows (8, 9): (1) Patients with persistent indications for PICC maintenance who require emergent catheter withdrawal secondary to critical complications; and (2) Patients who continue to need PICC access but suffer inadvertent device disengagement attributable to procedural (e.g., insecure fixation, dressing failure) or behavioral factors (e.g., infant movement, accidental traction by caregivers or staff).

Insertion: Performed by trained NICU nurses or physicians under sterile conditions using full aseptic technique. Catheter type: 1.9Fr single-lumen polyurethane catheter. Tip location confirmed by x-ray to be in the SVC/IVC. Maintenance: Dressings changed only when clinically indicated (soiled, loose, or compromised) to minimize dislodgement risk, following NANN neonatal guidelines (10). Catheter flushed with 0.9% saline before and after use. Patency maintained with heparinized saline (0.25–0.5 U/mL based on infant weight) locks after each use. Routine Removal: Upon completion of therapy, if malfunction persisted despite troubleshooting, or if complications (e.g., central Line Associated Bloodstream Infections, symptomatic thrombosis) met predefined criteria for removal.

This study was approved by the Ethics and research Committee of the Third Military Medical University Southwest Hospital, Chongqing, China (KY2024233), and all research procedures were conducted according to the principles of the Declaration of Helsinki. All data were fully anonymized and the requirement for informed consent was waived.

### 2.2 Data collection

We extracted neonatal demographic characteristics from medical records, including sex, gestational age, birth weight (g), delivery mode (categorized as eutocia or cesarean section), and perinatal status (including meconium-stained amniotic fluid and 5 min Apgar score). Through literature review, we additionally identified and collected potential UR-associated parameters: invasive mechanical ventilation (IMV) status, catheter insertion site (upper extremity vein/lower extremity vein), vasoactive medication use, and comorbidities [including hypercholanemia (HCA), pneumonia, necrotizing enterocolitis (NEC), and sepsis]—all assessed prior to PICC removal. Laboratory parameters within 5 days before removal were also recorded, comprising white blood cell count (WBC), absolute neutrophil count (ANC), platelet count (PLT), D-dimer (D-Di), and fibrinogen (Fib) levels. Data were extracted from electronic medical records. Cases with any missing data for the variables included in the final analysis were excluded (complete-case analysis was used).

### 2.3 Definitions of comorbidities

HCA: HCA in neonates is defined as serum total bile acid (TBA) level >40 μmol/L, often associated with symptoms like jaundice or liver dysfunction, excluding physiological elevations (11). Pneumonia: Neonatal pneumonia is diagnosed based on a combination of clinical signs (e.g., tachypnea >60 breaths/min, retractions, grunting, apnea, increased respiratory support), consistent radiographic findings (e.g., infiltrates, consolidations, or pleural effusions), and laboratory markers of infection [e.g., elevated C-reactive protein (CRP), white blood cell count abnormalities, or positive cultures from blood or tracheal aspirates] (12). NEC: NEC is diagnosed and staged according to modified Bell's criteria, with definite NEC defined as ≥Stage IIA (characterized by clinical signs like abdominal distension and bloody stools, plus radiographic evidence of pneumatosis intestinalis or portal venous gas, without perforation) (13). Sepsis: Neonatal sepsis is defined as a systemic inflammatory response to infection, with proven sepsis requiring a positive blood culture plus clinical signs (e.g., temperature instability, apnea, lethargy, tachycardia, hypotension) consistent with infection, often necessitating a full course of antimicrobial therapy (14).

### 2.4 Development and assessment of the model

Potential predictors were initially identified through univariate logistic regression analysis. Variables with *P* < 0.05 in the univariate analysis were considered candidates and entered into a multivariable logistic regression model. To derive a parsimonious model and mitigate overfitting, we employed a backward stepwise variable selection procedure using the Akaike Information Criterion (AIC) as the elimination criterion. Variables retained in the final model after this process (*P* < 0.05) were used to establish the predictive model for PICC-UR. With 55 events and 5 final predictors, the events-per-variable ratio was 11:1, which is above common recommended thresholds. Continuous variables (WBC, PLT, Fib) were initially assumed to have a linear relationship with the logit(*P*) and were visually checked using partial residual plots. To ensure the stability and interpretability of the final model, multicollinearity among the selected predictors was assessed using the Variance Inflation Factor (VIF); all VIFs were below 2.0, indicating no substantial multicollinearity. The nomogram was constructed by scaling each regression coefficient from the final multivariate logistic regression to a 0–100-point range. The total points were derived by summing the points assigned to each variable, corresponding to the predicted probability of PICC-UR.

Multicollinearity among the final predictors was assessed using the Variance Inflation Factor (VIF), and all VIFs were below 2.0, indicating no substantial collinearity.

The model's discrimination was evaluated using the area under the receiver operating characteristic curve (AUC) and the concordance index (C-index). Calibration was assessed using calibration curves and the Hosmer-Lemeshow test. Clinical utility was evaluated via decision curve analysis (DCA) and clinical impact curves (CIC). To rigorously assess model performance and correct for overoptimism, internal validation was performed using 1,000 bootstrap resamples. This process provides a bias-corrected estimate of the model's predictive accuracy and robustness.

### 2.5 Statistical analysis

All statistical analyses were performed using SPSS Statistics (version 26.0; IBM Corp., Armonk, NY, USA) and R software (version 4.1.2; R Foundation for Statistical Computing, Vienna, Austria). Continuous variables were presented as mean ± standard deviation (SD) for normally distributed data or median with interquartile range (IQR) for non-normally distributed data. Categorical variables were expressed as frequency (percentage). Group comparisons were conducted using independent samples t-test (normal distribution), Mann–Whitney *U*-test (non-normal distribution), or Chi-square test (categorical variables), as appropriate. Statistical significance was defined as a two-tailed *P*-value < 0.05 for all analyses.

## 3 Results

### 3.1 Risk factors for PICC-UR

**Figure 1:**
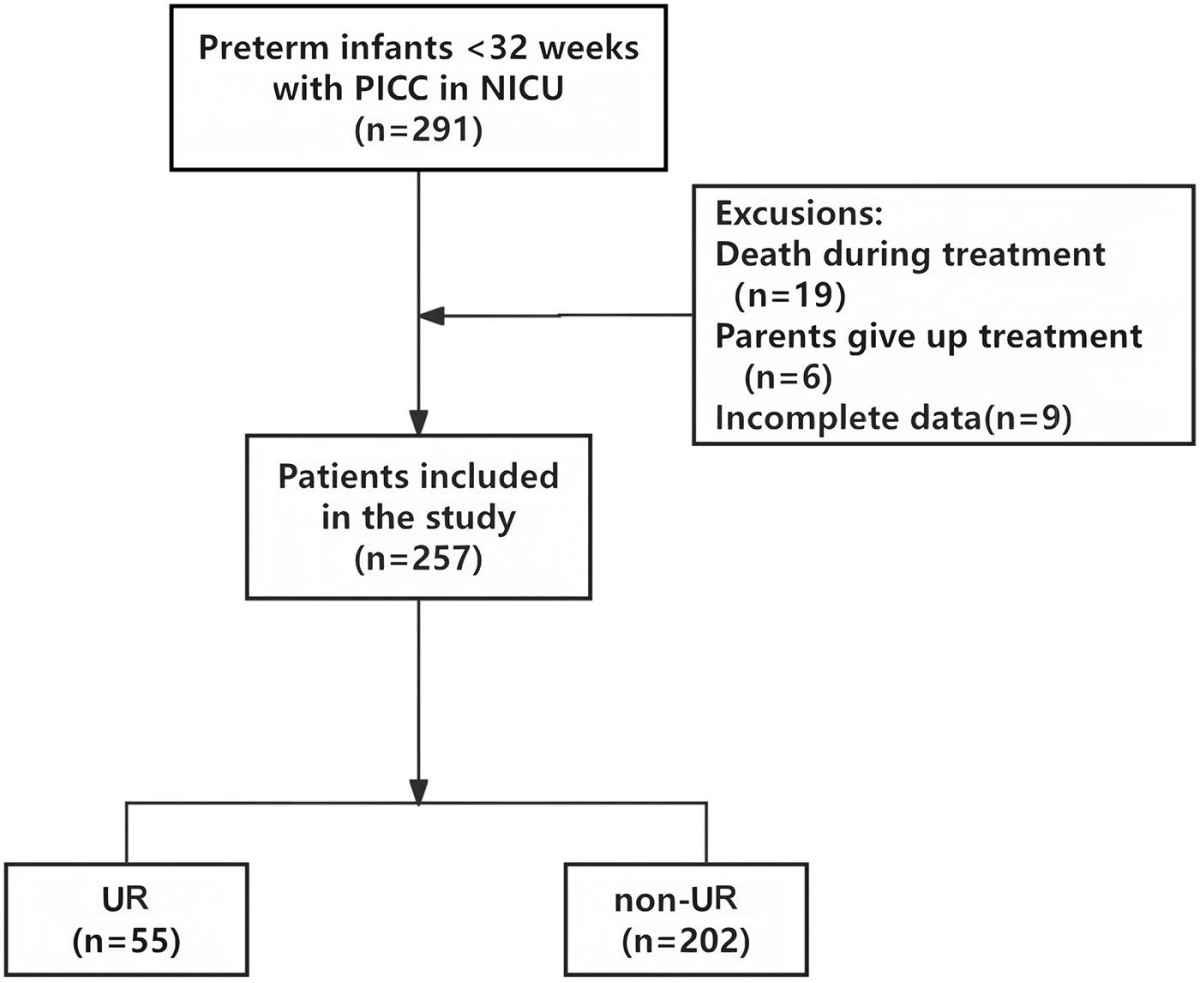
Flow chart for patient selection. PICC, peripherally inserted central catheters; NICU, neonatal intensive care unit; UR, unplanned removal.

Clinical data were retrospectively collected from 291 neonatal patients at Southwest Hospital of Third Military Medical University. 34 cases were excluded based on predefined exclusion criteria, resulting in 257 eligible participants for final analysis (Figure 1). Among these eligible infants, 55 experienced UR during intravenous therapy, yielding an incidence rate of 21.4% (55/257). These patients were assigned to the UR group (*n* = 55), while the remaining 202 comprised the non-UR group.

The comparative characteristics between groups are presented in Table 1. The UR group demonstrated significant differences in catheter insertion site, vasoactive medication administration, concurrent hypercholanemia, and sepsis incidence compared to the non-UR group. Furthermore, laboratory parameters including WBC, ANC, PLT, and Fib level showed statistically significant intergroup differences (*P* < 0.05). Notably, the indwelling duration of PICC was significantly shorter in the UR group than in the non-UR group (15.00 ± 5.40 vs. 31.26 ± 9.17).

**Table 1:** Comparison of baseline clinical characteristics, laboratory indicators, comorbidities and other parameters between the non-UR group and the UR group.

Variable	Non-UR group (*n* = 202)	UR group (*n* = 55)	*Z/t/χ^2^*	*P*
General data				
Sex, male, *n* (%)	107 (53.0)	24 (43.6)	1.507	0.220
Gestational age (weeks)	30.15 (28.40,31.20)	30.30 (29.00,31.20)	−0.224	0.823
Birth weight (g)	1,314.43 ± 297.46	1,260.91 ± 318.57	1.165	0.245
Delivery mode, eutocia, *n* (%)	61 (30.2)	22 (40.0)	1.900	0.168
Perinatal condition				
MSAF, *n* (%)	8 (4.0)	2 (3.6)	0.000	1.000
Apgar 5 min	10.00 (10.00,10.00)	10.00 (9.00,10.00)	−0.934	0.350
Laboratory metrics				
WBC (×10^9^/L)	10.33 (7.94,12.60)	12.29 (9.61,16.41)	−3.768	0.000
ANC (×10^9^/L)	3.56 (2.60,5.18)	5.82 (3.64,7.27)	−4.532	0.000
PLT (×10^9^/L)	264.00 (199.00,343.25)	347.00 (251.00,498.00)	−4.405	0.000
D-Di (mg/L)	1.03 (0.48,2.00)	1.31 (0.67,2.21)	−1.677	0.094
Fib (g/L)	1.96 (1.40,2.50)	1.20 (0.81,1.99)	−4.314	0.000
Comorbidities				
HCA, *n* (%)	77 (38.1)	41 (74.5)	23.099	<0.001
Pneumonia, *n* (%)	140 (69.3)	37 (67.3)	0.083	0.773
NEC, *n* (%)	19 (9.4)	6 (10.9)	0.111	0.739
Sepsis, *n* (%)	26 (12.9)	15 (27.3)	6.687	0.010
Other parameters				
IMV, *n* (%)	124 (61.4)	38 (69.1)	1.101	0.294
Insertion Site, upper extremity vein, *n* (%)	95 (47.0)	39 (70.9)	9.878	0.002
Vasoactive Drug Use, *n* (%)	99 (49.0)	37 (67.3)	5.787	0.016
PICC Dwell Time (days)	31.26 ± 9.17	15.00 ± 5.40	17.534	<0.001

Continuous data are presented as mean ± standard deviation or median (interquartile range) as appropriate; Categorical data are presented as *n* (%). Comparisons were made using the independent samples *t*-test, Mann–Whitney *U*-test, or Chi-square test, as appropriate. MSAF, meconium-stained amniotic fluid; WBC, white blood cell; ANC, absolute neutrophil count; PLT, platelet; D-Di, D-Dimer; Fib, fibrinogen; HCA, hypercholanemia; NEC, necrotizing enterocolitis; IMV, invasive mechanical ventilation; PICC, peripherally inserted central catheters.

The direct clinical reasons for unplanned PICC removal were meticulously recorded and are summarized in Supplementary Table S1. Mechanical causes, notably catheter displacement/migration (*n* = 20, 36.4%) and accidental traction/removal (*n* = 12, 21.8%), constituted the most frequent reasons for UR (63.7% overall). Complications, such as occlusion (*n* = 13, 23.6%) and suspected infection (*n* = 5, 9.1%), accounted for 36.3% of cases.

### 3.2 Screening for predictive factors

Potential predictors of PICC-UR were initially identified through univariate logistic regression analysis. As detailed in Supplementary Table S2, variables with a significance level of *P* < 0.05 in the univariate analysis included (insertion site, WBC, PLT, Fib, HCA, and sepsis).

Subsequently, these identified variables were incorporated into a multivariate logistic regression model to identify independent predictors. Multivariable logistic regression analysis identified five independent predictors of PICC-UR: insertion site (upper extremity vein) [*P* = 0.022, odds ratio (OR) 2.488, 95% confidence interval (CI) 1.14–5.431], WBC (*P* = 0.002, OR: 1.152, 95% CI: 1.054–1.26), PLT (*P* = 0.000, OR: 1.007, 95% CI: 1.004–1.011), Fib (*P* = 0.001, OR: 0.491, 95% CI: 0.321–0.753), and concomitant HCA (*P* = 0.000, OR: 4.905, 95% CI: 2.243–10.729). The final regression model (Table 2) was expressed by the equation: Ln (P/1-P) = 0.912*Insertion Site (Upper Extremity Vein = 1, Lower Extremity Vein = 0)−0.711*Fib + 0.142* WBC + 0.007*PLT + 1.590*HCA (No = 0, Yes = 1)−6.391.

**Table 2:** Predictors of UR in preterm infants with gestational age <32 weeks.

Predictor	B	SE	Wald χ^2^	*P*	OR (95% CI)
Insertion Site (upper extremity vein)	0.912	0.398	5.237	0.022	2.488 (1.14–5.431)
WBC	0.142	0.046	9.639	0.002	1.152 (1.054–1.26)
PLT	0.007	0.002	18.401	0.000	1.007 (1.004–1.011)
Fib	−0.711	0.218	10.643	0.001	0.491 (0.321–0.753)
HCA	1.590	0.399	15.863	0.000	4.905 (2.243–10.729)
Constants	−6.391	1.106	33.413	0.000	–

OR, odds ratio; CI, confidence interval; WBC, white blood cell; PLT, platelet; Fib, fibrinogen; HCA, hypercholanemia.

Restricted cubic spline analysis suggested a potential non-linear relationship for WBC, but it was retained as a linear term in the final model to preserve clinical practicality and interpretability of the nomogram.

Notably, although clinical variables such as sepsis and vasoactive drug use were significant in univariate analysis, they were not retained as independent predictors in the final multivariable model. This suggests that their association with PICC-UR may be mediated through the more objective and quantifiable laboratory parameters (WBC, PLT, Fib) or HCA that were retained, which capture the underlying inflammatory, coagulant, or metabolic dysregulation.

### 3.3 Risk prediction nomogram development

**Figure 2:**
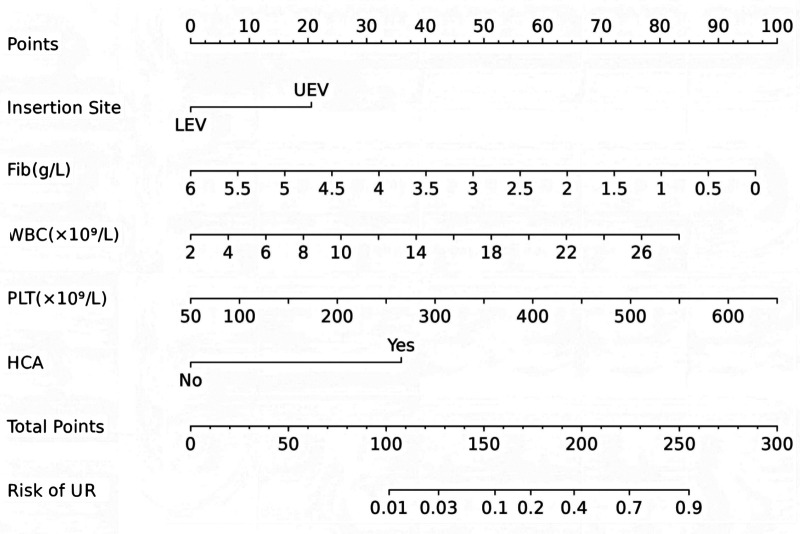
Nomogram of PICC-UR. Fib, fibrinogen; WBC, white blood cell; PLT, platelet; HCA, hypercholanemia.

According to the results of multivariable logistic regression analysis, the following factors were associated with PICC-UR: insertion site, WBC, PLT, Fib and HCA. These five factors were included in the prediction model, and a nomogram was created to visualize the results of the regression analysis (Figure 2).

### 3.4 Validation of nomogram

**Figure 3:**
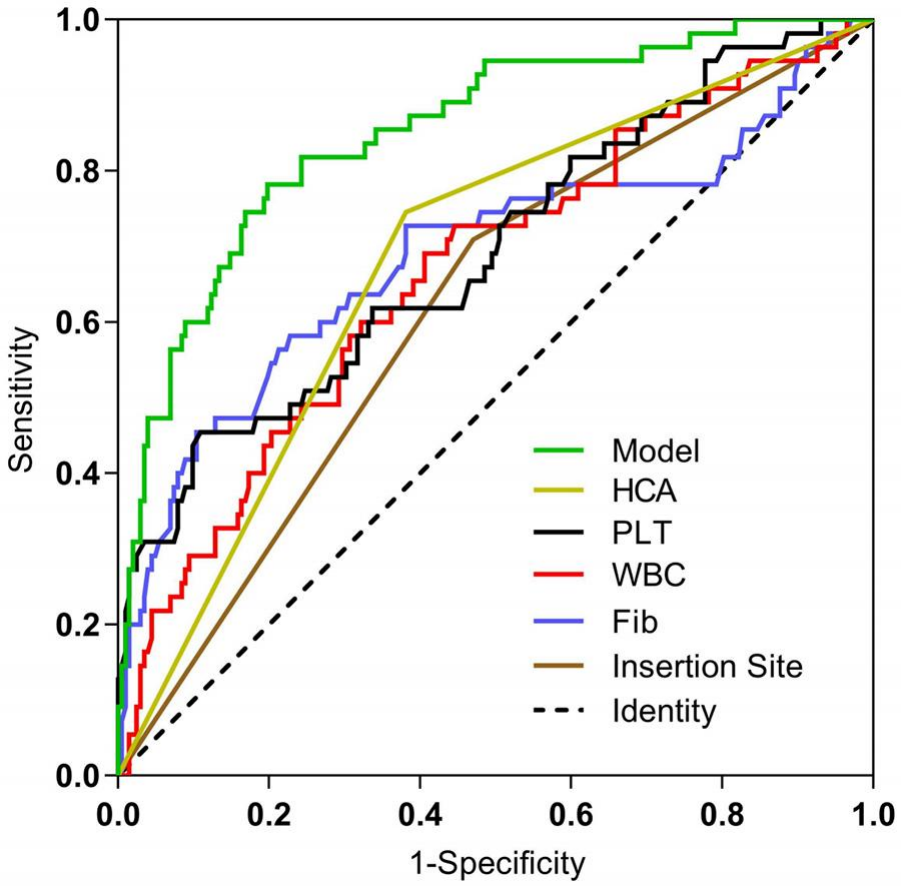
Comparison of ROC curves for each predictor and prediction model. Fib, fibrinogen; WBC, white blood cell; PLT, platelet; HCA, hypercholanemia.

The ROC curves and corresponding AUC values for insertion site, WBC, PLT, Fib, HCA, and the predictive model were 0.619, 0.666, 0.694, 0.690, 0.682, and 0.851, respectively, as shown in Figure 3, Table 3. The optimal cutoff values for each parameter, determined by maximizing Youden's index, along with their associated sensitivity, specificity, positive predictive value, and negative predictive value are presented in Table 3. Statistically significant differences (*P* < 0.05) were observed between the AUC values of the predictive model and each individual predictor.

**Table 3:** Comparison of the prediction effects of each independent predictor and prediction model of PICC-UR.

Predictor	AUC	95% CI	*P*	cutoff values	Sensitivity	Specificity	PPV	NPV
Insertion Site	0.619	0.538–0.701	0.007	–	70.9	53.0	29.1	87.0
WBC	0.666	0.583–0.749	<0.001	11.035	69.1	59.4	31.7	87.6
PLT	0.694	0.611–0.777	<0.001	393.5	45.5	89.1	53.2	85.7
Fib	0.690	0.599–0.781	<0.001	1.31	58.2	77.2	41.0	87.2
HCA	0.682	0.604–0.760	<0.001	–	74.5	61.9	34.7	89.9
Predictive Model	0.851	0.793–0.909	<0.001	0.239	78.2	80.2	51.8	93.1

WBC, white blood cell; PLT, platelet; Fib, fibrinogen; HCA, hypercholanemia.

**Figure 4:**
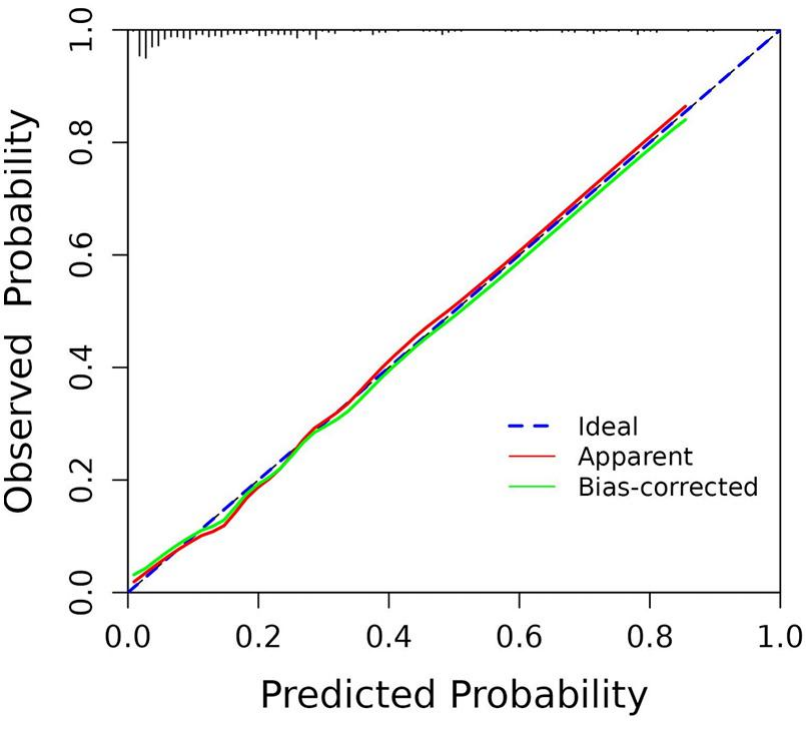
Calibration curve for predicting the probability of PICC-UR.

By internally validating the accuracy of the prediction model using the Bootstrap resampling technique, a C-index of 0.827 was obtained, with a strong fit between the original and corrected curves, demonstrating the effectiveness of the prediction model (Figure 4). However, these performance estimates are derived from internal validation. The model's generalizability and true predictive accuracy require confirmation through external validation in independent prospective cohorts.

### 3.5 Net benefit, and clinical impact of the nomogram

**Figure 5:**
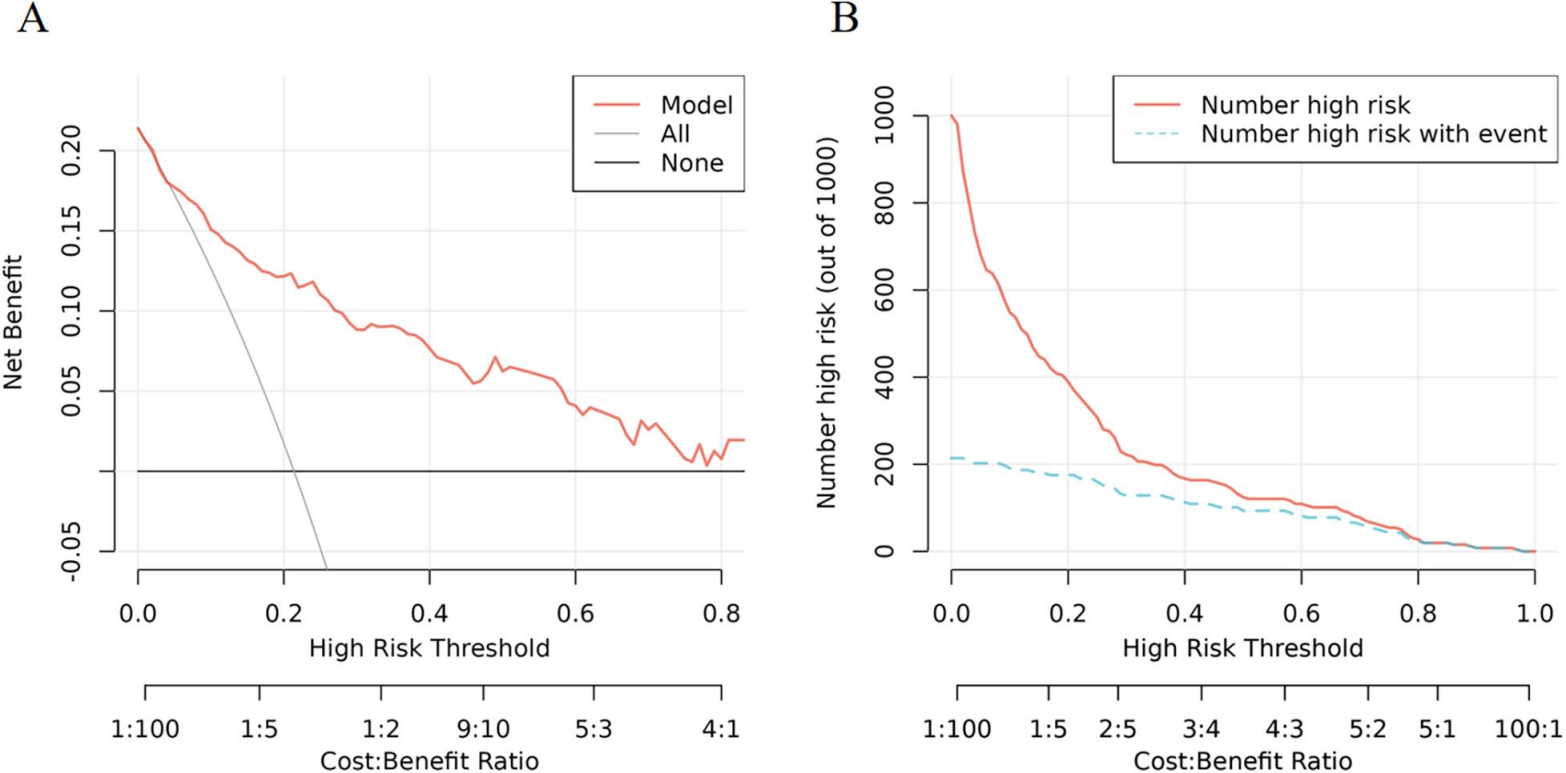
Validation and evaluation of prediction models. **(A)** Decision curve analysis in prediction of PICC-UR. **(B)** Clinical impact curve in prediction of PICC-UR.

The DCA showed significantly good net benefit in the predictive model (Figure 5A), and CIC visually indicated that nomogram conferred high clinical net benefit and confirmed the clinical value of the nomogram (Figure 5B).

## 4 Discussion

This study is the first to construct and validate a predictive nomogram for PICC- UR in preterm infants, identifying five independent predictors: insertion site, WBC, PLT, Fib, and HCA. The associations we observed, particularly for the laboratory markers and HCA, are novel and should be interpreted as exploratory. The following pathophysiological discussions are proposed to provide biological context and generate hypotheses for future research. In contrast to previous studies that primarily examined other PICC-related complications such as infection or thrombosis, our model specifically focuses on UR, highlighting the unique role of inflammatory and metabolic markers (e.g., HCA) in preterm infants and addressing the gap in preterm infant-specific risk prediction tools.

The selection between upper and lower extremity veins for PICC placement in preterm infants requires careful balance between anatomical considerations, technical success rates, and complication profiles. While upper limb veins are traditionally preferred in clinical practice due to providing a shorter, more direct path to the superior vena cava (15), our study found that selecting lower limb veins as the puncture site can reduce UR rates. Neonatal mobility challenges persist despite optimal catheter fixation, with upper limb positioning demonstrating 2.2-rib-space catheter migration during minor arm movements, potentially compromising tip stability (16). Lower extremity access, particularly through the relatively straight and robust great saphenous vein, facilitates direct inferior vena cava access with enhanced positional stability in non-ambulatory neonates (17). Cumulative evidence indicates superior first-attempt success rates, reduced catheter malposition rates, and lower unplanned removal incidence with lower extremity PICC (17–20). Furthermore, for mechanically ventilated preterm infants, catheter tip stability might be influenced by changes in intrathoracic pressure, potentially contributing to migration over time.

This study is the first to identify WBC, PLT, and Fib as independent predictors of PICC-UR in preterm infants with gestational age <32 weeks. Elevated WBC, often indicative of systemic inflammation, may compromise catheter stability by increasing vascular permeability and local tissue edema (21). This inflammatory state, commonly driven by conditions like sepsis or NEC, can alter the local vascular environment and endothelial integrity, thereby predisposing the catheter to dislodgement or removal due to associated complications. A meta-analysis (22) of adult cancer patients demonstrated that WBC >9.5 × 10^9^/L is an independent risk factor for PICC-related venous thrombosis, and thrombus formation could indirectly precipitate catheter displacement or removal. Furthermore, neonatal infections (e.g., sepsis), strongly associated with elevated WBC, may exacerbate catheter dislodgement risk through endothelial adhesion molecule activation (23). Clinically, for infants presenting with elevated WBC levels, intensified monitoring for and aggressive treatment of underlying infections (such as sepsis or NEC) which may be driving the leukocytosis should be prioritized. This includes more frequent blood cultures, earlier antibiotic intervention when clinically indicated, and enhanced infection control measures to address the inflammatory source that may destabilize catheter position. Although the platelet counts in both groups were generally within the normal neonatal range, the significantly higher values in the UR group suggest that even physiological variations may influence thrombotic risk and catheter stability. Increased PLT might influence UR may via dual potential mechanisms: (1) platelet activation promotes microthrombus formation, leading to mechanical obstruction at the catheter tip or vascular spasm (24); and (2) platelet-derived proinflammatory factors might amplify local inflammation, exacerbating perivascular tissue edema and catheter dysfunction (25). Thus, elevated PLT may signify a pro-thrombotic and pro-inflammatory milieu that challenges catheter patency and stability. Hao N et al. (26) identified elevated PLT as an independent risk factor for PICC thrombosis and occlusion, which is consistent with our findings demonstrating its significant association with UR in preterm infants. For infants with elevated platelet counts, although proactive pharmacological intervention is not standard practice, clinicians should maintain heightened vigilance for early signs of catheter dysfunction, such as sluggish flow rates or increased resistance during flushing, which might indicate impending thrombosis. This warrants implementation of more frequent flushing protocols, earlier line clearance strategies, and systematic assessment of catheter patency. Reduced Fib levels, reflecting hepatic impairment or consumptive coagulopathy (27), may impair fibrin sheath formation around the catheter, destabilizing intravascular fixation and increasing displacement risk during infant movement. Recent studies report an inverse correlation between Fib levels and catheter-related thrombosis risk (28); post-thrombotic pain or hemodynamic alterations may further trigger accidental removal. In cases of hypofibrinogenemia, particularly when levels fall below 1.0 g/L, consideration should be given to fibrinogen replacement therapy, as correction of critically low levels may improve tissue integrity and catheter sealing, thereby reducing displacement risk.

Beyond their independent effects, the synergistic interaction of these three biomarkers may potentially exacerbate UR risk through a theoretical “inflammation-coagulation-metabolism” axis. For instance, WBC-elevated inflammatory responses can potentially activate platelets (increasing PLT aggregation) while simultaneously depleting Fib, and the resultant hypofibrinogenemia further compromises vascular endothelial stability—a pathophysiological cascade mirroring sepsis-induced coagulopathy in neonates (29). This speculative interplay underscores the need for integrated clinical interventions: dynamic multi-parameter monitoring (e.g., infection control for leukocytosis, anticoagulation strategies for thrombocytosis, and Fib replacement therapy) to mitigate compounding risks. For high-risk infants identified by elevated nomogram scores, implementing enhanced securement techniques becomes crucial. This includes utilization of sutureless securement devices, application of reinforced transparent dressings, more frequent dressing inspections (every 24–48 h vs. standard 72 h intervals), and meticulous attention to limb positioning to minimize mechanical stress on the catheter.

Our study identified HCA as the strongest independent predictor of PICC-UR in preterm infants. This finding suggests a potential link between bile acid metabolism and PICC complication pathophysiology, which may operate through mechanisms distinct from classical inflammatory or thrombotic pathways. The observed association could be explained by several interconnected and speculative biological hypotheses: (1) Direct vascular toxicity—hydrophobic bile acids may disrupt endothelial tight junctions, increasing vascular permeability (30) and potentially compromising catheter stability; (2) Hemodynamic influence—activation of TGR5 receptors by bile acids might alter venous tone or flow patterns in preterm neonates (31); and (3) Systemic inflammatory interplay—HCA may URegulate neutrophil adhesion molecules and enhance platelet-derived proinflammatory mediators, thereby potentially synergizing with elevated WBC and PLT to aggravate perivascular inflammation (32, 33). Therefore, HCA may represent a unique metabolic risk factor that contributes to UR through direct vascular effects, altered hemodynamics, and/or potentiation of inflammation. The particular vulnerability of preterm infants to these effects might be attributable to hepatobiliary immaturity—resulting in reduced bile acid conjugation—and delayed gut colonization, which prolongs exposure to unmetabolized bile acids (34, 35). For infants presenting with HCA, active management of cholestasis remains essential. This may include ursodeoxycholic acid administration, optimization of enteral nutrition using medium-chain triglycerides, and phototherapy in cases with significant hyperbilirubinemia. In light of the elevated risk identified in these infants, more frequent assessment of catheter position and reinforced monitoring protocols could be considered.

The primary strength and clinical utility of this nomogram lie in its ability to provide a quantitative, individualized risk assessment early after PICC insertion. This allows clinicians to prioritize nursing care, tailor monitoring intensity, and implement preemptive strategies for infants identified as high-risk, potentially reducing the incidence of a common and disruptive complication. By stratifying patients based on their calculated risk scores, healthcare teams can allocate resources more efficiently, focusing intensive monitoring and preventive interventions on those most likely to experience unplanned removal, while maintaining standard care protocols for lower-risk patients.

## 5 Limitations and future directions

This study has several important limitations. First, the prediction model was developed and internally validated using data from a single center. Although bootstrap resampling provides a robust internal assessment and corrects for over-optimism, the generalizability of the nomogram and its reported performance require confirmation through external validation in independent, prospective, and preferably multi-center cohorts. This is an essential step prior to considering clinical implementation. Second, our retrospective single-center design may introduce potential for unmeasured confounders. We addressed this by systematically collecting clinically plausible variables previously associated with PICC complications from the literature and providing detailed definitions of our institutional protocols to enhance transparency and reproducibility. Third, our analysis focused on the first PICC insertion per infant to avoid clustering effects, which may not capture the full spectrum of PICC-related events in infants requiring multiple insertions. Fourth, unmeasured confounders such as specific catheter materials and variations in nursing protocols may influence outcomes. We have addressed this limitation by adding a detailed description of our standardized PICC insertion and maintenance protocols in the Methods section to contextualize our findings. Fifth, our primary outcome, unplanned removal (UR), is a composite endpoint that encompasses distinct mechanisms, including mechanical causes (e.g., displacement, traction) and complication-related causes (e.g., occlusion, suspected infection). While this reflects the pragmatic clinical need to predict any event leading to premature catheter loss, the predictive factors for different UR subtypes may differ. Our current sample size precludes robust subgroup analysis to identify specific predictors for each subtype; future studies with larger cohorts are warranted to investigate this heterogeneity. Sixth, infants who died prior to planned PICC removal were excluded from the analysis. This exclusion was necessary to avoid confounding, as the terminal physiological state could profoundly influence laboratory parameters and catheter status, and the removal event in this context does not represent the preventable outcome our model aims to predict. However, this criterion may have introduced selection bias by systematically excluding the most critically ill infants. Consequently, our model's predictions may be most accurate for the population of preterm infants who survive the acute critical phase requiring a PICC. Its performance in predicting UR among the most hemodynamically unstable infants, who are at potentially higher risk for all complications, requires further evaluation.

The foremost priority for future research is the external validation of this nomogram in diverse populations. Future multicenter prospective studies should externally validate this nomogram to evaluate its transportability, refine risk thresholds, and assess its real-world clinical impact. Additional research priorities include event-based analyses of all PICC insertions per infant, mechanistic investigations of HCA's role in catheter complications, analyses to identify predictors for specific UR subtypes, and intervention trials testing targeted prevention strategies based on this predictive model to improve outcomes in preterm infants.

## 6 Conclusion

This study developed and validated a novel nomogram for predicting PICC-UR in preterm infants with GA < 32 Weeks, integrating five independent risk factors: insertion site, WBC, PLT, Fib and HCA. This nomogram enables clinicians to implement targeted interventions based on individualized risk assessment, potentially improving catheter longevity and reducing the clinical burden associated with unplanned PICC removal in this vulnerable population.

## Data Availability

The raw data supporting the conclusions of this article will be made available by the authors, without undue reservation.
