# Supplementary material for: Development and validation of a nomogram for predicting unplanned PICC removal in preterm infants with gestational age <32 weeks
Source: Front Pediatr. 2026 Feb 12;14:1725396. doi: 10.3389/fped.2026.1725396 (PMC12935902; doi:10.3389/fped.2026.1725396)
Supplement: Supplementary file 1 [file Supplementaryfile1.docx]

**Supplementary Table 1.** Direct Clinical Reasons for PICC-UR in Preterm Infants (GA <32 weeks)

| Reason for Unplanned Removal (UR) | Category | *n (%) (N=55)* |
| --- | --- | --- |
| **Mechanical Causes** |  |  |
| Catheter displacement / migration | Mechanical | [20] (36.4%) |
| Accidental traction or removal | Mechanical | [12] (21.8%) |
| Catheter damage or leakage | Mechanical | [3] (5.5%) |
| **Complications** |  |  |
| Occlusion / Inability to aspirate | Complication | [13] (23.6%) |
| Suspected catheter-related bloodstream infection | Complication | [5] (9.1%) |
| Symptomatic thrombosis | Complication | [1] (1.8%) |
| Phlebitis / Thrombophlebitis | Complication | [1] (1.8%) |

PICC, peripherally inserted central catheter; UR, unplanned removal; GA, gestational age.

**Supplementary Table 2.** Univariate logistic regression analysis of factors associated with PICC unplanned removal

|  | *B* | *SE* | *Waldχ^2^* | *P* | *OR(95% CI)* |
| --- | --- | --- | --- | --- | --- |
| Sex | 0.375 | 0.306 | 1.498 | 0.221 | 1.455(0.798~2.652) |
| Gestational age | -0.099 | 0.079 | 1.574 | 0.210 | 0.906(0.777~1.057) |
| Birth weight | -0.001 | 0.001 | 1.354 | 0.245 | 0.999(0.998~1.000) |
| Apgar 5min | -0.101 | 0.138 | 0.535 | 0.465 | 0.904(0.691~1.184) |
| Delivery mode | -0.432 | 0.315 | 1.884 | 0.170 | 0.649(0.350~1.203) |
| MSAF | -0.089 | 0.806 | 0.012 | 0.912 | 0.915(0.189~4.438) |
| IMV | 0.341 | 0.326 | 1.095 | 0.295 | 1.406(0.743~2.662) |
| Insertion Site | 1.010 | 0.329 | 9.443 | 0.002 | 2.745(1.442~5.228) |
| Vasoactive Drug Use | 0.760 | 0.320 | 5.643 | 0.018 | 2.139(1.142~4.004) |
| D-Di | 0.065 | 0.091 | 0.504 | 0.478 | 1.067(0.892~1.275) |
| Fib | -0.771 | 0.210 | 13.453 | 0.000 | 0.462(0.306~0.698) |
| WBC | 0.141 | 0.038 | 13.832 | 0.000 | 1.152(1.069~1.241) |
| ANC | 0.179 | 0.054 | 10.883 | 0.001 | 1.196(1.075~1.330) |
| PLT | 0.007 | 0.001 | 25.332 | 0.000 | 1.007(1.005~1.010) |
| HCA | 1.559 | 0.342 | 20.808 | 0.000 | 4.754(2.433~9.289) |
| Pneumonia | -0.094 | 0.325 | 0.083 | 0.773 | 0.910(0.481~1.722) |
| NEC | 0.165 | 0.495 | 0.111 | 0.739 | 1.179(0.447~3.113) |
| Sepsis | 0.932 | 0.369 | 6.390 | 0.011 | 2.538(1.233~5.227) |

OR, odds ratio; CI, confidence interval; MSAF，meconium-stained amniotic fluid；IMV, invasive mechanical ventilation; D-Di, D-Dimer; Fib, fibrinogen; WBC，white blood cell；ANC，absolute neutrophil count；PLT，platelet；HCA, hypercholanemia; NEC, necrotizing enterocolitis; PICC, peripherally inserted central catheters;
